# Tumefactive demyelinating CNS lesion in a 60-year-old woman with familial Mediterranean fever

**DOI:** 10.1007/s10354-021-00893-z

**Published:** 2021-11-03

**Authors:** Constanze Trostel, Kornelia Laichinger, Till-Karsten Hauser, Sebastian Saur, Markus Krumbholz, Jörg Henes, Ulf Ziemann, Markus C. Kowarik

**Affiliations:** 1 Department of Neurology & Stroke, Eberhard-Karls University Tübingen, Tübingen, Germany; 2 Department of Neuroradiology, University of Tuebingen, Tübingen, Germany; 3 Department of Internal Medicine II, Eberhard-Karls-University Tübingen, Tübingen, Germany; 4 Hertie-Institute for Clinical Brain Research, Eberhard-Karls University Tübingen, Tübingen, Germany; 5 Department of Neurology, Klinikum rechts der Isar, Technical University of Munich, Munich, Germany

**Keywords:** Familiar Mediterranean fever, Demyelination, Tumefactive CNS lesion, Multiple sclerosis, MR spectroscopy, Familiäres Mittelmeerfieber, Demyelinisierung, Tumefaktive ZNS-Läsion, Multiple Sklerose, MR-Spektroskopie

## Abstract

We here report on a 60-year-old woman with familial Mediterranean fever (FMF) who developed cognitive impairment 16 years after initial diagnosis. On MRI, a new extensive white matter lesion in the right frontal lobe with mild local mass effect but without contrast enhancement was detectable and classified as a tumefactive lesion. Additional MR spectroscopy showed markedly increased choline levels accompanied by a significant lactate peak, highly suggestive of a low-florid demyelinating process. Although diffuse central nervous system (CNS) lesions have been described in single FMF cases, tumefactive lesions have not been observed in FMF patients without concomitant multiple sclerosis. In summary, this case highlights rare differential diagnoses of atypical, inflammatory CNS lesions and the clinical utility of MR spectroscopy.

## Abstract (de)

Die Autoren berichten in dem vorgestellten Fall von einer 60-jährigen Frau mit vorbekanntem familiärem Mittelmeerfieber (FMF), die 16 Jahre nach Diagnosestellung subakut kognitive Defizite entwickelt hatte. In der Bildgebung mittels Magnetresonanztomographie (MRT) zeigte sich als mögliche Erklärung dafür eine neue, frontal rechts gelegene Läsion mit leichtem Masseneffekt, aber ohne Kontrastmittelaufnahme, die als tumefaktive Läsion eingestuft wurde. Eine ergänzende MR-Spektroskopie zeigte einen deutlich erhöhten Cholin-Peak zusammen mit einem Laktat-Peak, was einen niedrigfloriden demyelinisierenden Prozess nahelegte. Auch wenn diffuse Läsionen des zentralen Nervensystems (ZNS) bei FMF vorbekannt sind, so wurden bislang tumefaktive Läsionen bei FMF ohne begleitende Multiple Sklerose nicht beschrieben. Zusammenfassend werden anhand dieses Falls seltene Differenzialdiagnosen von atypischen ZNS-Läsionen und der klinische Nutzen der MR-Spektroskopie hervorgehoben.

## Introduction

Familial Mediterranean fever (FMF) is a hereditary autoinflammatory disease characterized by fever episodes that are often accompanied by painful peritonitis, pleuritis, arthritis, cutaneous manifestations, and—less commonly—inflammation of the central nervous system (CNS) [1, 2]. FMF is caused by autosomal recessive or autosomal dominant mutations in the *MEFV* (Mediterranean fever) gene. *MEFV* gene mutations have been shown to interfere with pyrin formation, leading to an increased activity of the proinflammatory cytokine interleukin‑1 beta (IL-1β), which results in autoinflammatory reactions with various presentations [1–3].

## Case report

### Medical history

We here report on a 60-year-old woman of Turkish origin who was diagnosed with FMF in 2003 due to recurrent fever episodes and detection of the homozygous *MEFV* M694V mutation. Initially, the patient described fever episodes every 3–4 months, often in combination with abdominal or diffuse pain that improved after colchicine treatment in 2003 (dosage between 1–4 × 0.5 mg/d). Subsequently, fever episodes were less frequent but several comorbidities including atrial fibrillation, depression, knee arthroplasty, and two curatively treated malignancies (thyroid cancer 2005 and breast cancer 2012, therapies not including cerebral radiotherapy) occurred. No direct relationship between the mentioned diseases and FMF was suspected, amyloidosis as a direct comorbidity of FMF [2] was ruled out by rectal biopsy in 2019.

### Neurological findings

In 2020, the patient was admitted to our neurologic clinic for diagnostic work-up of unspecific neurological symptoms and abnormal MRI findings. The patient reported a slightly impaired memory, poor concentration, and absentmindedness during everyday activities in combination with headaches and more frequent fever episodes every 2–4 weeks during the last 14 months. Clinical examination was without pathologic findings except cognitive impairment (Montreal Cognitive Assessment test 16/30 points).

**Fig. 1:**
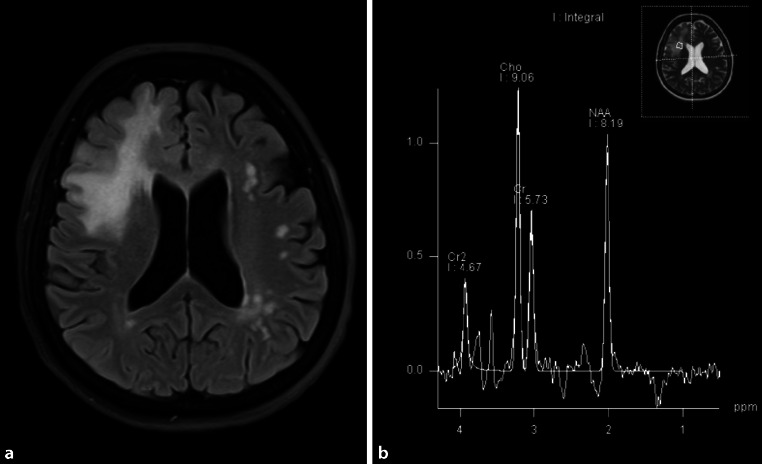
Tumefactive, demyelinating central nervous system lesion in MRI and MR spectroscopy transverse fluid-attenuated inversion recovery (FLAIR) image. Image (**a**) shows extensive T2 hyperintensity of the right frontal lobe with a maximum range of 6.2 cm. No associated contrast enhancement or diffusion restriction could be detected (disease exacerbation > 12 months before current MRI). Image (**b**) shows MR spectroscopy using chemical shift imaging with an increase of choline (*Cho*), normal N‑acetyl aspartate (*NAA*), and a characteristic negative double peak at 1.3 ppm, representing lactate. The marked increase of choline accompanied by the lactate peak is highly suggestive of a low-florid demyelinating process

Due to recurrent episodes with headaches, external cerebral MRI scans had been performed in 2002 and 2008 and shown unspecific white matter lesions. Until 2019, no other neurological symptoms or relapses were reported, and the patient received no other immunomodulatory treatments than colchicine. After the occurrence of new cognitive symptoms, a follow-up MRI scan in October 2019 showed a new, ill-defined white matter lesion in the right frontal lobe, with mild local mass effect but without contrast enhancement. In the cerebral MRI from December 2020, the lesion was essentially constant in size and appearance (Fig. 1a). The additional MR spectroscopy showed markedly increased choline levels accompanied by a lactate peak, highly suggestive for a low-florid demyelinating process (Fig. 1b). Due to the lesion size > 2 cm, the slight mass effect, and the demyelinating character, we classified the right-sided frontal lobe lesion as a tumefactive demyelinating lesion [4]. Further diagnostic procedures including brain biopsy or FET-PET were discussed with the patient but not pursued, also following the patient’s choice.

Cerebrospinal fluid (CSF) examination including cell count; protein, glucose, and IgG index; MRZ reaction; and oligoclonal IgG bands (OCB) did not show any pathological findings. There was no evidence of infectious disease (JCV PCR, HIV, hepatitis, syphilis, and Lyme serology all negative). Further laboratory investigations, including anti-aquaporin‑4, anti-MOG, and thyroid autoantibodies showed normal results. Electrophysiologic diagnostics showed delayed visual evoked potentials on the right side and delayed sensitive evoked potentials on the upper extremities (not tolerated at the lower limbs); motor evoked potentials were within normal limits.

## Discussion

A number of case reports [5, 6] have already described demyelinating CNS lesions and the diagnosis of multiple sclerosis (MS) in association with FMF and *MEFV *mutations [1, 7, 8]. In our patient, the diagnostic criteria for MS were not fulfilled due to the lack of defined clinical relapses. Furthermore, typical CSF features, such as CSF-specific OCBs, were not detectable. As possible differential diagnoses for the tumefactive lesion, infectious diseases as well as a brain tumor appeared to be unlikely due to the stable presentation over 1 year and the MR spectroscopy results which showed a marked increase of choline, no relevant reduction of the N‑acetyl aspartate peak, and a significant lactate peak, suggestive for a demyelinating process. Consequently, the described lesions including the tumefactive lesion were classified as demyelinating lesions in the context of the FMF diagnosis and treatment was changed to an anti-IL‑1 therapy (anakinra). As a limitation of this case, it has to be pointed out that there is no final evidence causally linking the tumefactive lesion to FMF. In follow-up, clinical symptoms and MRI remained stable, with a slight improvement of cognitive deficits. Although the exact pathophysiology underlying CNS lesion formation in FMF remains unclear, the increased activity of IL-1β has been suggested to facilitate a proinflammatory milieu that triggers endothelial dysfunction and favors the development of autoreactive lymphocytes [7].

## Conclusion

In summary, we here present an unusual case of FMF with demyelinating CNS lesions including one tumefactive lesion. Although diffuse CNS lesions have been mentioned in single FMF cases [5, 9], tumefactive lesions have not been described outside of patients with concomitant MS. This case highlights rare differential diagnoses of atypical CNS lesions and the clinical utility of MR spectroscopy.

## Abbreviations

CNS: Central nervous system
CSF: Cerebrospinal fluid
FMF: Familial Mediterranean fever
IL-1β: Interleukin‑1 beta
JCV: John Cunningham virus
*MEFV*: Mediterranean fever gene
MOG: Myelin oligodendrocyte glycoprotein
MRI: Magnetic resonance imaging
MRZ: Measles, rubella, and varicella zoster
MS: Multiple sclerosis
OCB: Oligoclonal IgG bands
PCR: Polymerase chain reaction
PET: Positron-emission tomography
